# Representation of In-Service Performance for Cable-Stayed Railway–Highway Combined Bridges Based on Train-Induced Response’s Sensing Data and Knowledge

**DOI:** 10.3390/s22093247

**Published:** 2022-04-23

**Authors:** Han-Wei Zhao, You-Liang Ding, Ai-Qun Li

**Affiliations:** 1 Key Laboratory of Concrete and Pre-Stressed Concrete Structures of the Ministry of Education, Southeast University, Nanjing 210096, China; wudizhw_0@126.com; 2 Beijing Advanced Innovation Center for Future Urban Design, Beijing University of Civil Engineering and Architecture, Beijing 100044, China; liaiqun@bucea.edu.cn

**Keywords:** structural health monitoring, live-load response, data-driven deterioration detection, structural dynamics, bridge

## Abstract

Real-time representation of the current performance of structures is an important task for perceiving potential danger in in-service bridges. Methods driven by the multisource sensing data of structural health monitoring systems are an effective way to achieve this goal. Due to the explicit zero-point of signals, the live load-induced response has an inherent advantage for quantitatively representing the performance of bridges. Taking a long-span cable-stayed railway–highway combined bridge as the case study, this paper presents a representation method of in-service performance. First, the non-stationary sections of train-induced response are automatically extracted by wavelet transform and window with threshold. Then, the data of the feature parameter of each non-stationary section are automatically divided into four cases of train load according to the calculational theory of bridge vibration under train effect and clustering analysis. Finally, the performance indexes for structural deformation and dynamics are determined separately, based on hierarchical clustering and statistical modeling. Fusing the real variability of massive data from monitoring and the knowledge of mechanics of theoretical calculations, accurate and robust indexes of bridge deflection distribution and forced vibration frequency are obtained in real time. The whole process verifies the feasibility of the representation of bridge in-service performance from massive multisource sensing data. The presented method, framework, and analysis results can be used as a reference for the design, operation, and maintenance works of long-span railway bridges.

## 1. Introduction

Perceiving the potential danger to a bridge is the primary reason for installing a structural health monitoring (SHM) system on a bridge [1,2,3]. With the development of testing, communication, and computer technology, multiple types of physical quantities of bridge input (load) and output (response) can be accurately obtained [4,5]. However, perceiving the potential danger to a structure is a comprehensive task; it is a process from measurement to evaluation [6]. After the accurate measurement of the bridge input–output behavior, the real-time state of the structure should be accurately evaluated to guide bridge operation and maintenance. The quantitative representation (i.e., description of objects by one or multiple features) of the bridge in-service performance using the knowledge of mechanics is the primary task for the evaluation of a bridge’s in-service state.

Traditionally, the representation of bridge performance is a forward problem with known cases of loading; it is generally based on calculation, simulation, or experiment. In the early stages, many scholars derive the indexes of the dynamic or capacity performance of the bridge based on the theory of classical mechanics [6,7,8,9,10]. With the wide application of numerical simulation, some scholars investigate the dynamic, capacity, and fatigue performance of bridges under load effects based on the results of finite element simulation [11,12,13]. Scholars who focus on local performance and failure modes study the bending and shearing capacity of the components and structures of bridges, based on interactive verification by experiments and simulations [14,15,16,17]. In recent years, some scholars have presented the real-time hybrid simulation method to analyze the performance of the whole structure of bridges [18,19,20]. However, due to the scale effect of experiments and the equivalent simplification of simulations, this research may not reflect the real performance of bridges.

Due to urgent needs for monitoring the current performance of existing bridges, there has been a renewed focus on the method of performance representation for non-initial state bridges under real, unknown, or random in-service loads based on field measurements. These methods involve a typical inverse problem [21], which is proposed to determine the current state of the bridge structure based on the load-induced response. Modal parameters of the structure identified by free-vibration without loads are the most common index of performance for bridge monitoring [22,23,24], and many frequency-based methods with high robustness to environmental effects have been proposed for detecting structural damage in bridges [25,26]. Measured displacement [27,28] and strain [29,30] data have also been used to assess bridge long-term performance. Because existing bridges are always subject to in-service loads, the feature parameters of the response under temperature, vehicle, train, and wind are used to establish the performance index [31,32,33,34,35]. Based on the calculated index of performance, methods of early warning for the potential danger of bridges in operation have been proposed [36,37,38,39,40]. Nevertheless, current methods of performance representation are not real-time and automatic enough for the operation and maintenance of bridges; their core parameters require experienced professionals’ determination and adjustment. The advantages of the massive data [41] from bridges’ SHM systems have not been fully exploited. The mining and evaluation methods using multi-type response data of bridges under various loading cases need to be studied [42].

The application of a modern SHM system for bridges is a technology involving fusion perception from multiple input–output parameters; it collaboratively measures different physical quantities using different types of sensors. This makes feasible the real-time [43] representation of the structural performance as driven by data. Taking a 1290 m cable-stayed railway–highway combined bridge as the case study, this paper proposes a representation method of in-service performance based on multisource sensing data and theoretical calculation. The advantage of SHM data in the analysis of structural real-time non-initial performance is exploited, and the knowledge of mechanics on the bridge vibration under a train’s effects is introduced. The indexes for representing the bridge deformation and dynamic performances are finally determined based on the massive data of train-induced non-stationary response.

## 2. Case Study Bridge and Sensor Deployment

**Figure 1:**
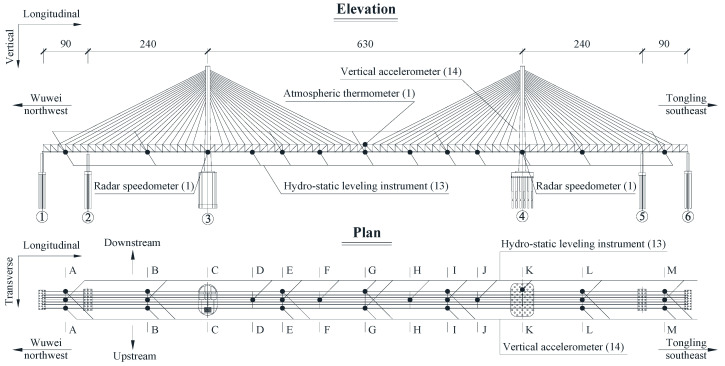
Deployment of monitoring sensors of the bridge (unit: meters).

The case study is a long-span cable-stayed railway–highway combined bridge on the Changjiang river in Anhui, China. As indicated in Figure 1, the total length of the bridge is 1290 m, with a continuous span configuration of (90 + 240 + 630 + 240 + 90) meters; the main span of the bridge is 630 m. This bridge adopts the structure type of three girder trusses along the transverse direction and three cable planes along the longitudinal direction. The center of the three main trusses of the girder is 17.1 m apart along the transverse direction. The height of the trusses of the girder is 15.5 m, and upper and a lower orthotropic box steel bridge decks are set for the highway and railway respectively. The lower layer of the girder consists of two 250 km/h high-speed railway tracks on the downstream and two 160 km/h fast railway tracks on the upstream. The upper layer of the girder consists of six lanes of highway. As of the date of the monitoring data, all highway lanes and the downstream high-speed railway tracks were opened to traffic, while the upstream fast railway tracks were not. The number of carriages of high-speed trains on these railway lines is only 8 and 16. Two bearings are set on each pier to support the girder; the downstream bearings of each pier resist the vertical and transverse motion, while the upstream bearings of each pier resist the vertical motion. The two towers are both 212 m high. A total of 8 longitudinal viscous dampers are set between two towers and the girder (near the bearings) to resist the longitudinal impact of velocity greater than 0.5 mm/s (e.g., earthquake or the braking force of trains and vehicles). The two towers and four piers of the bridge are set on pile foundations with different depths.

**Figure 2:**
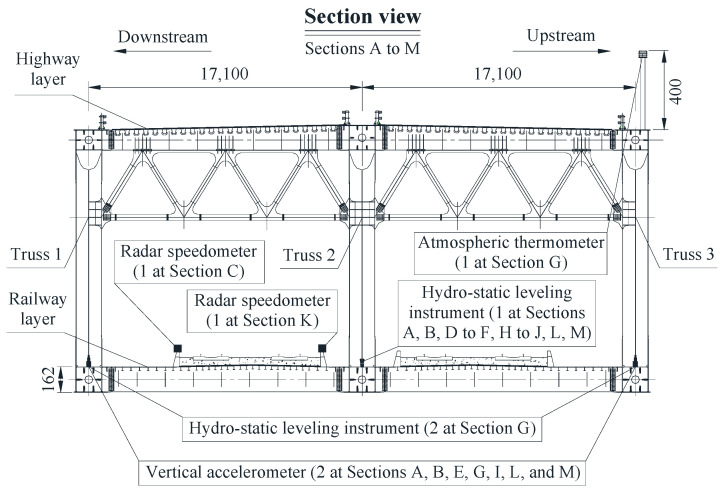
Sensor location of each monitoring section (unit: millimeters).

Figure 1 and Figure 2 indicate the deployment of monitoring sensors for the representation of bridge performance in this paper. The deformation and dynamic performances are two important items for the bridge during operation. Hence, the deflection and acceleration responses are selected as study objects to represent the bridge deformation and dynamic performance. A hydrostatic leveling instrument is used to measure the deflection (namely the quasi-dynamic vertical displacement) of the bridge. In each of Sections A, B, D–F, H–J, L, and M, one hydrostatic leveling instrument is set at the joint of the middle-truss rod and lower bridge deck. In the Section G, two hydrostatic leveling instruments are set at the joints of two side-truss rods and the lower bridge deck. Considering that only the two downstream high-speed railway tracks were open to traffic before the last day of the data used in this paper, all deflection data of Section G take the measured data of the downstream hydrostatic leveling instrument of Section G. In Section K, a hydrostatic leveling instrument is set on Pier 4 as the datum point for the whole hydrostatic leveling system. Two vertical accelerometers are set at the joints of two side-truss rods and the lower bridge deck in each of Sections A, B, E, G, I, L, and M. An atmospheric thermometer is set at the top of the truss in the Section G to measure the atmospheric temperature and humidity. Two radar speedometers are set near the two downstream high-speed railway tracks in Sections C and K to measure the speed of each traversing of trains on the bridge; correctly setting the measuring angle of the radar speedometer can easily accomplish the task of just measuring the speed from the train traversing its installed track. The sensing details of the above types of sensors are indicated in Table 1. The data transmission and power supply of all sensors uses the wired method. A data room is set for storing data near the bridge site. The sensors′ deployment and optimization are the important parts of the SHM [44]. The sensor deployment of the case study bridge is optimized by the dual driving of mechanics′ knowledge and monitoring experience, but it is not the focus of this paper and is not described here.

## 3. Feature Extraction of Train′s Effects from Multiple Types of Sensing Data

Whether the structural response (output) under the in-service load (input) exceeds the limit is the intuitive reflection of structural safety. To represent the deformation and dynamic performance, the input–output mechanism of the bridge under in-service loads should be investigated. Since some accidental failures (e.g., power cut) of the sensor or SHM system may cause the drift of signal during long-term monitoring, the initial point (namely the zero-point) of temperature-induced deflection cannot be determined effectively [34,45]. Hence, the temperature-induced deflection may not be a good index for evaluating the in-service performance of the bridge. The live load-induced response is more useful for representing the bridge deformation and dynamic performance.

**Figure 3:**
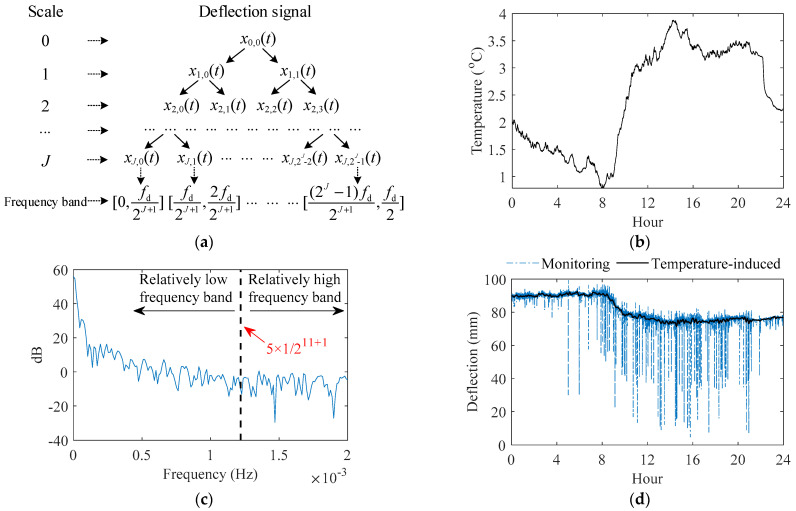
Determination of temperature-induced deflection by wavelet transform: (**a**) decomposition tree of signal by *J*-scale wavelet packet; (**b**) temperature data; (**c**) frequency spectrum of temperature data; (**d**) deflection data and its temperature-induced part.

During operation of railway–highway combined bridges, in-service loads such as temperature, trains, vehicles, and wind act on the structure at the same time. If it is a cross-sea bridge whose girder is a steel box girder, the high-frequency response may also come from the effect of wind [46]. However, the girder of the case study bridge of this paper is a steel truss girder, and the effect of wind is not obvious. To accurately analyze and discuss the live load′s effects on the deflection, the temperature′s effects should be normalized. The measured deflection signal can be decomposed to different deflection parts in different frequency bands by a packet of multiple wavelet transform. The temperature-induced deflection is in the relatively low frequency band, and the live load-induced deflection (including the vehicle-induced and train-induced deflection) is in the relatively high frequency band. The decomposition scale of the wavelet transform can be determined by the frequency spectrum of the signal [37,47]. Assume that the signal frequency of the temperature data is mainly concentrated at [0, *f*_t_], and the relatively low and high frequency bands can be determined. According to the definition of *Nyquist* frequency, the frequency band available for the deflection signal with a sampling frequency of *f*_d_ is [0, *f*_d_/2]. As indicated in Figure 3a, the 0th decomposed sequence of the *J*th layer using the *J*-scale wavelet packet belongs to [0, *f*_d_/2*^J^*^+1^]. The optimal decomposition scale of the wavelet transform can be determined by selecting an appropriate *J*, which makes *f*_d_/2*^J^*^+1^ slightly larger than *f*_t_ and *f*_d_/2*^J^*^+2^ smaller than *f*_t_. Then, the temperature-induced deflection (the low-frequency part of deflection) can be considered as the decomposed part of the lowest frequency band from the obtained results by the decomposition of the wavelet transform. Taking the data of January 6 in one year as an example, Figure 3 indicates the decomposition processes of the temperature-induced deflection of Section G based on the above steps; the mother wavelet utilized is *Symlets 6*. Figure 3a indicates the decomposition tree of the signal using the *J*-scale wavelet packet. Figure 3b indicates the temperature data of this day. The parameter *J* is determined to be 11 according to the frequency spectrum of temperature data of this day shown in Figure 3c. As indicated in Figure 3d, the temperature-induced deflection can be accurately obtained. Comparing Figure 3b,d, it can be found that the signal trend of temperature-induced deflection presents negative correlation with the signal trend of atmospheric temperature on the same day; when one value is taken for every five values of temperature-induced deflection data, the correlation coefficient between the temperature-induced deflection data of compressed sampling frequency and temperature data is −0.8537.

**Figure 4:**
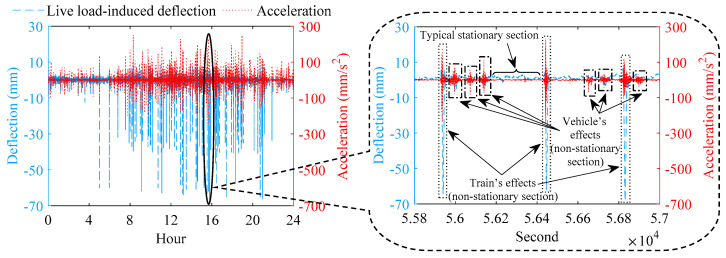
Live load-induced deflection and acceleration and their characteristics of time series signal.

When the temperature-induced deflection is obtained, the live load-induced deflection (namely the high-frequency part of the deflection) can be gained, which equals the raw measurement deflection minus the temperature-induced deflection. Figure 4 indicates the live load-induced deflection and acceleration at the middle of the main span of the bridge (Section G) on January 6 in one year. The acceleration data in Figure 4 are from the downstream vertical accelerometer of Section G, and considering that only the two downstream high-speed railway tracks are open to traffic, all acceleration data in the following text of this paper are the measured data of the downstream vertical accelerometers. As indicated in Figure 4, the time series signal of the live load-induced deflection and acceleration consists of the stationary section and the non-stationary section. The non-stationary section is the bridge response under each traversing of train or vehicle, which characterizes the feature of live-load response. In Figure 4, the dotted boxes are train-induced responses, and the dash-dotted boxes are vehicle-induced responses. Compared with the non-stationary section of vehicle-induced deflection, the non-stationary section of train-induced deflection has a higher amplitude; minimum values of all non-stationary sections of train-induced deflection of Section G are over −16 mm, while minimum values of all non-stationary sections of vehicle-induced deflection of Section G do not reach −14 mm. The train′s effect on the deflection is more significant than the vehicle′s effect on the deflection. Compared with the non-stationary section of vehicle-induced acceleration, the non-stationary section of train-induced acceleration has shorter length, which is 0.7 to 0.9 times that of the non-stationary section of vehicle-induced acceleration. For representing of bridge in-service performance, the matching relationship between the non-stationary section of train-induced deflection, the non-stationary section of train-induced acceleration, train speed, and temperature on the time coordinate should be established, which requires the time of measurement for the data from various sensors to be recorded.

Based on the characteristics of the signal of train-induced and vehicle-induced response, the train-induced response and the vehicle-induced response can be effectively distinguished; then, the corresponding non-stationary sections can be automatically extracted. First, the sections of the time series signal of live load-induced deflection are cut out automatically using sliding windows, checking whether the absolute value of the minimum of each cut-out section of live load-induced deflection is higher than the empirical threshold of height. Then, if the absolute value of the minimum of some cut-out sections of live load-induced deflection is higher than the empirical threshold of height, we check whether the data on train speed at the same time period is at the normal range of speed of the high-speed train (the data of train speed will equal zero when no train traverses the bridge); which radar speedometer the train speed data belongs to needs to be recorded. Finally, if the data of train speed from one of the radar speedometers is at the normal range of speed of the high-speed train (each radar speedometer only records the speed of train in its installed track), the corresponding cut-out sections of the live load-induced deflection, acceleration, train speed, and temperature at the same periods can be judged as the train-induced sections. Each cut-out section records its time; if several adjacent sections are judged to be non-stationary sections, and they have the repeated minimum value of the train-induced deflection, only the middle one is extracted.

After the computer automatically extracts non-stationary sections of the train-induced response, it can be spot-checked manually based on the above characteristics of signal of train-induced and vehicle-induced deflection and acceleration shown in Figure 4. Heavy vehicles over 55 tons are not permitted on the bridge; the weight of each vehicle on the bridge is less than the weight of one train carriage. What is more, if a heavy vehicle traverses at the same time of a passing heavy train, the proposed method of performance presentation based on massive monitoring data can eliminate the influence of the small amount of individual randomness as presented in this situation.

The amplitude of the train-induced non-stationary deflection in Sections A, B, L, and M is mostly below 15 mm, which is much smaller than that in Sections D–J. Hence, monitoring data in Sections A, B, L, and M will not be presented and discussed in this paper.

**Figure 5:**
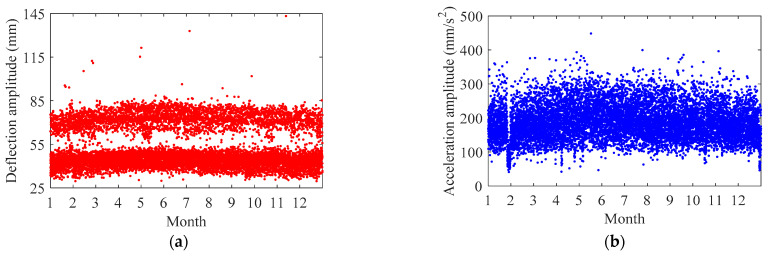
Amplitude of each non-stationary section of train-induced response at Section G in one year: (**a**) deflection; (**b**) acceleration.

**Figure 6:**
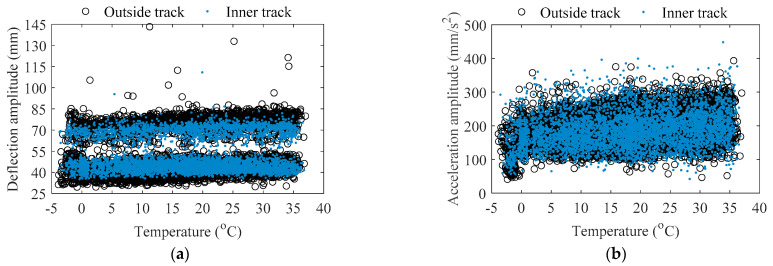
Amplitude of train-induced non-stationary response versus temperature at Section G: (**a**) deflection; (**b**) acceleration.

Figure 5 indicates the amplitude of each non-stationary section of train-induced response arranged by order of the traversing of the train at Section G in one year. In this paper, the amplitude of train-induced deflection is defined as the maximum minus minimum of each non-stationary section of train-induced deflection, and the amplitude of train-induced acceleration is defined as the maximum of absolute value of each non-stationary section of train-induced acceleration. It can be found in Figure 5 that both the amplitude of train-induced deflection and the amplitude of train-induced acceleration present characteristics that the intermediate data (i.e., the data in summer) are slightly higher than the data at both ends (i.e., the data in winter). Based on the track information from radar speedometers, the amplitude data of each non-stationary section of train-induced response can be divided into two cases: the case of outside track and the case of inner track; the scatter plots of the amplitude of train-induced non-stationary response versus the temperature at Section G in the two cases are indicated in Figure 6. The amplitude of train-induced acceleration and the amplitude of train-induced deflection of the outside track case shows slightly positive correlation with temperature (the correlation coefficients are 0.2422 and 0.2025, respectively). However, the amplitude of train-induced deflection of the inner track has no correlation with temperature (the correlation coefficient is 0.0666). The differences of the train-induced deflection amplitude′s temperature correlation in the two cases of outside and inner tracks may be because the train-induced deflection amplitude in the outside track case consists of the torsion behavior of the girder, while the inner track does not. It should be noted that, as indicated in Figure 5a and Figure 6a, whether it is the outside track case or the inner track case, the amplitude data of train-induced deflection naturally divide into two clusters, which are in different heights. This phenomenon should be deeply investigated by the mechanism of the bridge vibration under the train′s effects.

## 4. Knowledge of Train-Induced Response from Theoretical Calculation

The classical theory of calculation for the vibration of short- and medium-span bridges under a train′s effect has been deeply investigated [7,8,9]. For bridges whose length is near or longer than the length of trains (e.g., the case study bridge; its first order of the vibration mode is the symmetrical vertical bending vibration of the girder with the natural frequency of 0.33 Hz [48]), their amplitude of train-induced response under high-speed trains is derived here to explain the phenomenon at the end of the previous section.

**Figure 7:**
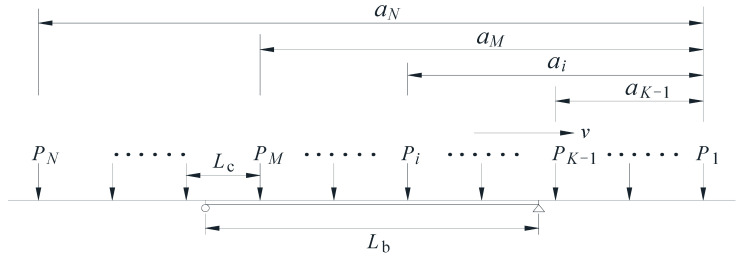
Simplified schematic diagram of the bridge subjected to a moving train.

*L*_b_ is the length of the main span of the bridge, and *L*_c_ is the length of a single train carriage. The loading model of the train traversing the bridge can be simplified as shown in Figure 7. *N* is the total number of train carriages, *K* − 1 is the last load leaving the bridge, and *M* is the last load on the bridge. *K* − 1 should be greater than or equal to 0, and *M* should less than or equal to *N*.

The analysis of the bridge vibration includes the first-order mode shape (vertical vibration) alone. It would be reasonable to expect the displacement of a point along the length of the bridge to vary with *x* and *t* as distinctly separate functions. We shall therefore assume that

(1) y(x,t)=ϕ(x)q(t)

where *y*(*x*,*t*) is the vertical displacement, *ϕ*(*x*) = sin(*nπx*/*L*_b_) is the first-order mode shape of the bridge vertical vibration, *q*(*t*) are the generalized co-ordinates that define the amplitude of vibration with time, *x* is the distance between the point and one of bridge ends, and *t* is a certain moment of bridge vibration.

Let the first and the last moving loads on the bridge be *P_K_* and *P_M_* at time *t* (Figure 7). If the first-order mode shape of the bridge vertical vibration is considered alone, the equation of vibration motion in generalized co-ordinates under a series of moving loads at a constant speed can be expressed as follows [7]:

(2) $$\ddot{q}(t)+2\xi \omega \dot{q}(t)+\omega^{2}q(t)=\frac{2}{mL_{b}}\sum_{i=K}^{M}\sin\frac{\pi (vt-a_{i})}{L_{b}}P_{i}$$

where *m* is the mass per unit length, *ω* is the first natural frequency (circular), *ξ* is the damping ratio, *v* is the velocity of moving loads (trains), *P_i_* is the *i*th moving load, *a_i_* = *L*_c_ (*i* − 1), which is the distance between moving loads *P*_1_ and *P_i_*, $\dot{q}(t)$ denotes the first derivative of *q*(*t*) with respect to time, and $\ddot{q}(t)$ denotes the second derivative of *q*(*t*) with respect to time. The solution of *q*(*t*) may be sought by first examining the steady vibration of the bridge under a single moving load and the transient vibration after the passage of the single load and subsequently using the principle of superposition. For the convenience of the superposition operation, consider the single moving load as the *i*th load in the moving load series, and let *t* = 0 be the time when the first moving load enters the bridge. Thus, the steady vibration caused by the *i*th moving load takes place when *a_i_*/*v ≤ t ≤ a_i_*/*v* + *L*_b_/*v*. Specializing Equation (2) for the *i*th single moving load only, the solution can be expressed as the combination of a steady term and a transient term, as follows:

(3) $$q(t)=\frac{\beta }{1-\beta^{2}}\sin\frac{\pi (vt-a_{i})}{L_{b}}-\frac{y_{\mathrm{st}}\beta }{1-\beta^{2}}e^{-\xi \omega (t-a_{i}/v)}\sin\omega (t-\frac{a_{i}}{v})$$

where *y*_st_ = *PL*3 b/48*EI* is static midspan deflection with moving load *P* acting on the midspan, *β* = *πv*/*ωL*_b_ with *ω* being the bridge natural frequency (circular), *E* denotes the elastic modulus of the bridge material and *I* denotes the second moment of area of the bridge cross section.

For the time *t* ≥ *t*^′^ = *a_t_*/*v* + *L*_b_/*v*, the transient vibration after the passage of the single (*i*th) moving load is

(4) $$q(t)=e^{-\xi \omega (t-t^{\prime })}\left[\frac{{\dot{q}}_{i}(t^{\prime })+\xi \omega q_{i}(t^{\prime })}{\omega }\sin\omega (t-t^{\prime })+q_{i}(t^{\prime })\cos\omega (t-t^{\prime })\right]$$

where $q_{i}(t^{\prime })$ and ${\dot{q}}_{i}(t^{\prime })$ are the displacement and velocity excited by the *i*th load. Substituting Equation (3) into the $q_{i}(t^{\prime })$ and ${\dot{q}}_{i}(t^{\prime })$ in Equation (4), Equation (4) can now be expressed as

(5) $$q(t)=e^{-\xi \omega (t-a_{i}/v-L_{b}/v)}\frac{y_{\mathrm{st}}\beta }{1-\beta^{2}}\sin\omega (t-\frac{a_{i}}{v}-\frac{L_{b}}{v})-e^{-\xi \omega (t-a_{i}/v)}\frac{y_{\mathrm{st}}\beta }{1-\beta^{2}}\sin\omega (t-\frac{a_{i}}{v})$$

According to Equations (3) and (5), the bridge response under a series of moving loads can be established using superposition. Consider a generic scenario of (*K* − 1), for which moving loads have traversed the bridge, while (*M* − *K* + 1) moving loads are acting on the bridge, and the bridge response at the time *a_K_*_−1_/*v* + *L*_b_/*v* ≤ *t* ≤ *a_K_*/*v* + *L*_b_/*v* can be expressed as

(6) $$\begin{array}{l} q(t)=\frac{y_{\mathrm{st}}}{1-\beta^{2}}\times \sum_{i=K}^{M}\sin\frac{\pi (vt-a_{i})}{L_{b}}-\frac{y_{\mathrm{st}}\beta }{1-\beta^{2}}\times \sum_{i=K}^{M}e^{-\xi \omega (t-a_{i}/v)}\sin\omega (t-\frac{a_{i}}{v})\\ -\frac{y_{\mathrm{st}}\beta }{1-\beta^{2}}\times \sum_{i=1}^{K-1}e^{-\xi \omega (t-a_{i}/v-L_{b}/v)}\sin\omega (t-\frac{a_{i}}{v}-\frac{L_{b}}{v})\\ -\frac{y_{\mathrm{st}}\beta }{1-\beta^{2}}\times \sum_{i=1}^{K-1}e^{-\xi \omega (t-a_{i}/v)}\sin\omega (t-\frac{a_{i}}{v}) \end{array}$$

In the Equation (6), the first two terms represent the bridge vibration caused by the acting of (*M* − *K* + 1) moving loads; the first one is a steady vibration term and the second one is a transient vibration term. The last two terms are the bridge transient vibration caused by the preceding (*K* − 1) moving loads.

Analyze the first term (the steady vibration response neglecting damping) of Equation (6) as follows:

(7) $$q_{\mathrm{sv}}(t)=\frac{y_{\mathrm{st}}}{1-\beta^{2}}\times \sum_{i=K}^{M}\sin\frac{\pi (vt-a_{i})}{L_{b}}$$

Analyze the last three terms (the transient vibration response neglecting damping) of Equation (6) as follows:

(8) $$q_{\mathrm{tv}}(t)=-\frac{y_{\mathrm{st}}\beta }{1-\beta^{2}}\times \sum_{i=1}^{M}\sin\omega (t-\frac{a_{i}}{v})-\frac{y_{\mathrm{st}}\beta }{1-\beta^{2}}\times \sum_{i=1}^{K-1}\sin\omega (t-\frac{a_{i}}{v}-\frac{L_{b}}{v})$$

The acceleration response of the transient vibration of bridge can be expressed as the second derivative of Equation (7):

(9) $${\ddot{q}}_{\mathrm{tv}}(t)=\frac{y_{\mathrm{st}}\omega^{2}\beta }{1-\beta^{2}}\times \sum_{i=1}^{M}\sin\omega (t-\frac{a_{i}}{v})+\frac{y_{\mathrm{st}}\omega^{2}\beta }{1-\beta^{2}}\times \sum_{i=1}^{K-1}\sin\omega (t-\frac{a_{i}}{v}-\frac{L_{b}}{v})$$

To facilitate the derivation, two expansions of the triangular series must be given:

(10) $$\sum_{i=1}^{m}\sin(a-ix)=\sum_{i=1}^{m}\left[\sin a\cos ix-\cos a\sin ix\right]$$

(11) $$\left\{ \begin{array}{l} \sum_{i=1}^{m}\sin ix=\sin\frac{mx}{2}\sin\frac{(m+1)x}{2}\csc\frac{x}{2}\\ \sum_{i=1}^{m}\cos ix=\sin\frac{mx}{2}\cos\frac{(m+1)x}{2}\csc\frac{x}{2} \end{array}\right.$$

Rewrite Equation (7) as

(12) $$q_{\mathrm{sv}}(t)=\frac{y_{\mathrm{st}}}{1-\beta^{2}}\times \left[\sum_{i=1}^{M}\sin\frac{\pi (vt-a_{i})}{L_{b}}-\sum_{i=1}^{K}\sin\frac{\pi (vt-a_{i})}{L_{b}}\right]$$

Substitute Equations (10) and (11) into Equation (12), and *a_i_* = *L*_c_ (*i* − 1); then, Equation (12) can be expressed as

(13) $$\begin{array}{l} q_{\mathrm{sv}}(t)=\frac{y_{\mathrm{st}}}{1-\beta^{2}}\times \frac{\sin\frac{ML_{c}}{2L_{b}}\sin\left[\frac{\pi vt}{L_{b}}-\frac{(M-1)L_{c}}{2L_{b}}\right]}{\sin\frac{L_{c}}{2L_{b}}}\\ -\frac{y_{\mathrm{st}}}{1-\beta^{2}}\times \frac{\sin\frac{KL_{c}}{2L_{b}}\sin\left[\frac{\pi vt}{L_{b}}-\frac{(K-1)L_{c}}{2L_{b}}\right]}{\sin\frac{L_{c}}{2L_{b}}} \end{array}$$

Merge the two terms in Equation (13), and it can be expressed as Equation (14).

(14) $$\begin{array}{l} q_{\mathrm{sv}}(t)=A_{\mathrm{sv}}\sin(\frac{\pi v}{L_{b}}t-\frac{(M+K-2)L_{c}}{4L_{b}}+\theta_{\mathrm{sv}}),\\ A_{\mathrm{sv}}=\frac{y_{st}}{(1-\beta^{2})\sin\frac{L_{c}}{2L_{b}}}\sqrt{A_{\mathrm{sv}1}^{2}+A_{\mathrm{sv}2}^{2}-2A_{\mathrm{sv}1}A_{\mathrm{sv}2}\cos\frac{(M-K)L_{c}}{2L_{b}}},\\ \tan\theta_{\mathrm{sv}}=-\frac{A_{\mathrm{sv}1}+A_{\mathrm{sv}2}}{A_{\mathrm{sv}1}-A_{\mathrm{sv}2}}\tan(\frac{(M-K)L_{c}}{4L_{b}}),A_{\mathrm{sv}1}=\sin\frac{ML_{c}}{2L_{b}},A_{\mathrm{sv}2}=\sin\frac{KL_{c}}{2L_{b}}. \end{array}$$

Substitute Equations (10) and (11) into Equation (9), and *a_i_* = *L*_c_ (*i* − 1); then, Equation (9) can be expressed as

(15) $$\begin{array}{ll} {\ddot{q}}_{\mathrm{tv}}(t)= & \frac{y_{st}\omega^{2}\beta }{1-\beta^{2}}\times \frac{\sin\frac{ML_{c}\omega }{2v}\sin\left[\omega t-\frac{(M+1)L_{c}\omega }{2v}\right]}{\sin\frac{L_{c}\omega }{2v}}\\ & +\frac{y_{st}\omega^{2}\beta }{1-\beta^{2}}\times \frac{\sin\frac{(K-1)L_{c}\omega }{2v}\sin\left[\omega t-\frac{\omega L_{b}}{v}-\frac{KL_{c}\omega }{2v}\right]}{\sin\frac{L_{c}\omega }{2v}} \end{array}$$

Merge the two terms in Equation (15), and it can be expressed as Equation (16).

(16) $$\begin{array}{l} {\ddot{q}}_{\mathrm{tv}}(t)=A_{\mathrm{tv}}\sin(\omega t-\theta_{\mathrm{tv}}),\\ A_{\mathrm{tv}}=\frac{y_{st}\omega^{2}\beta }{(1-\beta^{2})\times \sin\alpha }\sqrt{A_{\mathrm{tv}1}^{2}+A_{\mathrm{tv}2}^{2}},\tan\theta_{\mathrm{tv}}=\frac{A_{\mathrm{tv}1}}{A_{\mathrm{tv}2}},\alpha =\frac{L_{c}\omega }{2v},\\ A_{\mathrm{tv}1}=\sin M\alpha \sin(M+1)\alpha +\sin(K-1)\alpha \sin(\frac{\omega L_{b}}{v}+K\alpha ),\\ A_{\mathrm{tv}2}=\sin M\alpha \cos(M+1)\alpha +\sin(K-1)\alpha \cos(\frac{\omega L_{b}}{v}+K\alpha ). \end{array}$$

**Figure 8:**
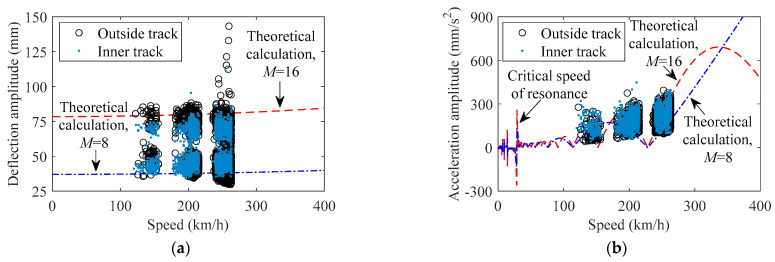
Amplitude of train-induced response versus train speed at Section G validated by theoretical calculation: (**a**) deflection; (**b**) acceleration.

According to Equations (14) and (16), the baseline of theoretical calculation for the correlation of train-induced deflection amplitude (*A*_sv_ in Equation (14)) versus train speed and train-induced acceleration amplitude (*A*_tv_ in Equation (16)) versus train speed can be obtained. Figure 8 indicates the results of the theory of calculation for the correlation of train-induced response amplitude versus train speed, and the corresponding data of monitoring is the same as in Figure 5 and Figure 6. The value of train speed of each point in Figure 8 takes the maximum of the train speed traversing the bridge (the train speed is basically constant in each traversing time). Due to the speed control during the operation of the high-speed train, the data of real speed of the train traversing the bridge are mainly at the three ranges of 120–160, 175–215, and 235–265 km/h. As seen in Figure 8, according to the length of the main span of the case study bridge and the size parameters of the electric multiple units of the China Railways High-Speed 3 [37], *L*_b_ (the length of bridge span) and *L*_c_ (the length of train carriage) take 630 and 24 m, respectively; the value of *ω* equals 0.33 Hz, which references the first vertical natural frequency of the bridge from the frequency spectrum analysis of the stationary section of the acceleration data, as well as design documents of the bridge [48]; dashed and dash-dotted curves in Figure 8a,b take *M* = 16 and *K* = 1, and *M* = 8 and *K* = 1, respectively; the equivalent *E* and *I* of the generalized beam depend on the design documents of the case study bridge, and the *P* depends on the weight of the fully loaded train carriage.

As indicated in Figure 8, the trend′s characteristic of the amplitude data of monitoring is basically in line with the theory of calculation; however, some variability exists in the monitoring results, which may be due to the randomness of the train carriage′s weight, track irregularities, difference in parameters of the train′s suspension system, or any other variation. It should be noted that the results of theoretical calculation well explain the phenomenon of two naturally different height clusters of train-induced deflection amplitude; this is due to the difference between 8 carriages and 16 carriages of the train traversing the bridge. Furthermore, the train-induced deflection amplitude will increase with the increasing of the number of the train carriages; readers can verify this using Equation (14). The critical speed of resonance will become higher as the span becomes shorter, and the resonance effect will be more obvious. This is why scholars studying bridge engineering [7,8] are generally concerned about the resonance performance of short- and medium-span railway bridges. The study object of this paper is the long-span bridge, so the critical speed of resonance will be not discussed here; readers can self-verify using Equation (16).

Based on the theory of calculation in this section, it can be found that the amplitude of train-induced deflection mainly depends on the steady vibration term of the bridge under the effect of trains, while the amplitude of train-induced acceleration mainly depends on the transient vibration term of the bridge under the excitation of trains. According to structural dynamics [49], transient vibration will be dissipated within a short period of time when the excitation disappears; the amplitude of train-induced acceleration may not be suitable for representing the dynamic performance of the bridge. The calculation model in this section is highly simplified, and it is only used for offering knowledge of mechanics to the data analysis. To investigate the dynamic behavior of the train-bridge system under the action of high-speed trains, a three-dimensional train-bridge coupled finite element model is more useful.

## 5. Representation of Bridge Deformation and Dynamic Performance

Combining the analysis results of Section 3 and Section 4, it can be found that the amplitude of train-induced deflection is a good feature parameter to represent the deformation performance of in-service bridges. On the other hand, considering that the frequency of train-induced forced vibration is always the focus of the design and maintenance for railway–highway combined bridges and high-speed-railway bridges [50], the frequency of train-induced forced vibration is chosen as the feature parameter to represent the dynamic performance of the bridge. The analysis in this section is based on the monitoring data within one year, but corresponding methods also can be used for data in the longer term.

### 5.1. Framework for Representation of Bridge In-Service Performance

**Figure 9:**
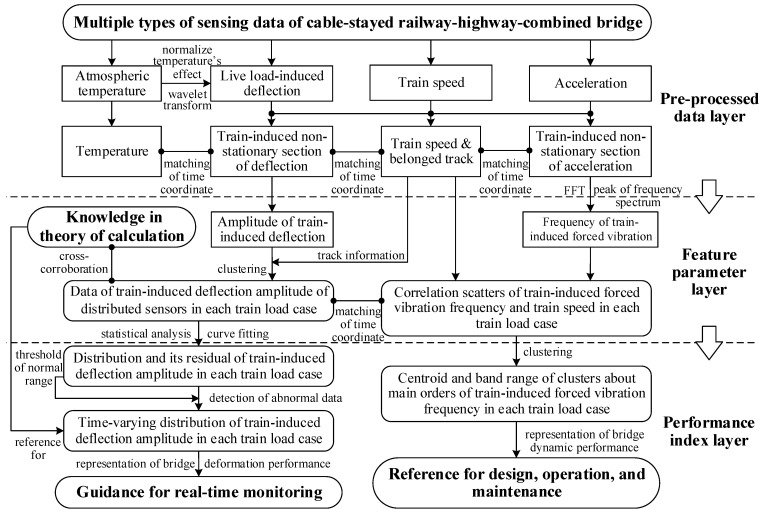
Framework of representation of in-service performance for cable-stayed railway-highway-combined bridge.

According to the above analysis, the right source of sensing data and feature parameters for representing the bridge deformation and dynamic performance was determined, and their representation methods based on multisource sensing data and theoretical calculation can thus be determined. An effective and practicable method is proposed as indicated in Figure 9. The framework of the proposed method consists of three layers, as follows.

In the pre-processed data layer (the first layer): Monitor the atmospheric temperature, train speed, deflection, and acceleration data of the bridge. Based on the frequency spectrum analysis of the temperature data, normalize the temperature′s effect of the deflection by wavelet transform. According to characteristics of the time series signal of live load-induced deflection, train speed, and acceleration, the train-induced non-stationary sections of deflection and acceleration are automatically distinguished and extracted. Establish the matching relationship of the time coordinate between the non-stationary section of train-induced deflection, the non-stationary section of train-induced acceleration, train speed, and temperature on the time coordinate.

In the feature parameter layer (the second layer): Calculate the train-induced deflection amplitude, the frequency of train-induced forced vibration, and the corresponding train speed. Based on track information from radar speedometers and the theory of calculation of the influence of 8 or 16 carriages, the feature parameters are divided into four cases of train load. Obtain the data of train-induced deflection amplitude of distributed sensors in each case of train load; obtain the correlation scatters of train-induced forced vibration frequency and train speed in each case of train load; and establish the matching relationship of the time coordinate between different feature parameters and different sensors.

In the performance index layer (the third layer): On the one hand, obtain clusters about the main orders of the frequency of train-induced forced vibration with the stability of temperature and train speed via clustering; calculate the centroid and band range of each cluster as the representation index of dynamic performance of the bridge. On the other hand, calculate the fitting curve of the distribution of the train-induced deflection amplitude in each case of train load from the data of multiple sensors; then, calculate the distribution residual between the fitting curve and the data of each traversing train; determine the statistical threshold of the normal range of the distribution of the amplitude of train-induced deflection to detect or clean the data with the signal abnormal; use a time-varying fitting curve to represent the long-term deterioration of deformation performance of the bridge.

Considering that the vibration in each cross section of the girder of the bridge is theoretically synchronized and coordinated [49], this paper only analyzes the frequency of train-induced forced vibration at the middle of the main span of the bridge (Section G). In contrast, the amplitude of train-induced deflection of the girder is at different level in each monitoring section, this paper chooses to analyze the distribution of the amplitude of train-induced deflection. It should be noted that the proposed method is not limited to use on cable-stayed railway–highway combined bridges; it can be promoted to any long-span railway bridge, with some adjustments and adaptations.

### 5.2. Classification of Feature Parameter about Different Cases of Train Load

Before obtaining the performance index, the feature parameters for the train-induced response of the bridge must be divided into four cases according to outside or inner track and 8 or 16 carriages. Then, the internal mechanism between the feature parameters of the bridge train-induced response and the performance indexes of in-service bridge can be linked. The data in the cases of the outside or inner track can be clearly distinguished by radar speedometers. However, the data in the cases of 8 or 16 train carriages can only be distinguished by fuzzy clustering.

Hierarchical clustering [51] is used for the fuzzy clustering task; the principle of hierarchical clustering based on agglomerative logic is to first treat all data points as independent data clusters; then, agglomerate the two clusters with the smallest distance until all data points are agglomerated to the same data cluster. In this paper, the *Euclidean* distance is used to define the distance between any two data points:

(17) $$d_{pq}=\left\|C_{p}-C_{q}\right\|=\sqrt{\sum_{j=1}^{F}{\left(c_{p,j}-c_{q,j}\right)}^{2}}$$

where ***C****_p_* and ***C****_q_* denote the vector of the data point *p* and *q* (the vector can be a one-dimensional or multi-dimensional vector), *F* denotes the dimension of the data point vector, and *c_p_*_,*j*_ and *c_p_*_,*j*_ denote the values of the *j*th dimension of the vectors ***C****_p_* and ***C****_q_*. As data points continue to be agglomerated, each data cluster will contain multiple data points. Hence, clusters in the process of hierarchical clustering also need a method of distance calculation, and this paper uses the Ward (incremental sum of squares) distance to calculate the distance between clusters:

(18) $$D(r,s)=\sqrt{\frac{2n_{r}n_{s}}{\left(n_{r}+n_{s}\right)}}{\left\|{\bar{x}}_{r}-{\bar{x}}_{s}\right\|}_{2}$$

(19) $${\bar{x}}_{r}=\frac{1}{n_{r}}\sum_{i=1}^{n_{r}}C_{ri}$$

where ${\bar{x}}_{r}$ and ${\bar{x}}_{s}$ denote the centroid of cluster *r* and cluster *s*, respectively (the calculation of centroid is defined as Equation (19)), *n_r_* denotes the number of data points in cluster *r*, ***C****_ri_* denotes the vector of *i*th data point in cluster *r*, and ${\left\|\cdot \right\|}_{2}$ denotes the calculation of *Euclidean* distance. Through the above processes, a hierarchical cluster tree that includes a series of binary trees can be determined; a (*Num* − 1)-by-3 linkage matrix is calculated, where *Num* is the numbers of data points. In each row of the linkage matrix, the first two elements are cluster indices linked in pairs to form a binary tree; the leaf nodes are numbered from 1 to *Num*; leaf nodes are the singleton clusters from which all higher clusters are built. Each newly formed cluster, corresponding to row (*I*, :), is assigned the index *Num* + *I*; the third element of the row is the distance of the clusters (*I*, 1) and (*I*, 2); the order of the rows in the linkage matrix are arranged in ascending order of (*I*, 3). In the hierarchical clustering, the output of the clustering results is controlled by calculating the inconsistency coefficient between each cluster:

(20) IC(k)=(LM(k)−MD(k))/SD(k)

where, ***LM***(*k*) denotes the (*k*th, 3) elements of the linkage matrix, ***MD***(*k*) denotes the mean of the distance values of all the linked clusters included in the calculation, and ***SD***(*k*) denotes the standard deviation of the distance values of all the linked clusters included in the calculation. The calculation of ***MD***(*k*) and ***SD***(*k*) can not only include the links of row *k*, but also a specified depth of levels in the hierarchical cluster tree. For example, if the depth is 10, there will be a maximum of 2^10^ clusters participating in the calculation of ***MD***(*k*) and ***SD***(*k*). The higher the depth is, the higher the nonlinear degree of hierarchical clustering is. When ***IC***(*k*) is larger than the preset threshold, the output of the clustering results stops.

**Figure 10:**
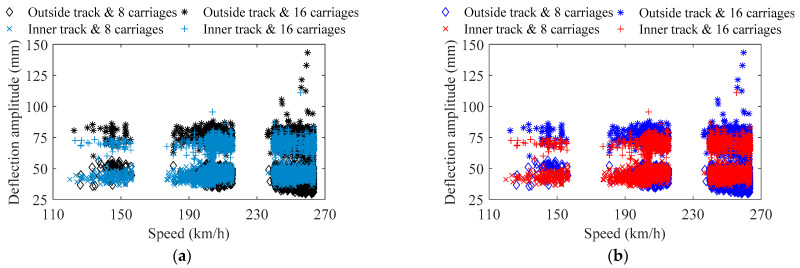
Cases for 8 and 16 carriages for train-induced deflection amplitude by clustering: (**a**) hierarchical clustering; (**b**) *k*-means clustering (*k* = 2).

Figure 10 indicates the clustering results of the train-induced deflection amplitude by hierarchical clustering, and a comparison is made by the *k*-means clustering [39]; the process of clustering only uses the data of train-induced deflection amplitude, but for cross-corroborating with the results of theoretical calculation in Figure 8a, the data for train speed is also given. The data of the outside track and inside track is clustered respectively, and the depth of hierarchical clustering is set to 10. As indicated in Figure 10, the data in the cases of the outside track and inside track are both divided into two sub-clusters; they can accurately describe the theoretical characteristics of the two types of cases of 8 and 16 carriages, and the clustering results by hierarchical clustering are consistent with the results of *k*-means clustering. For data points close to the boundary of the two clusters, a small amount of data may be misclassified, and as the amount of data accumulates, this error will be reduced to a low level.

According to the clustering results and the track information from radar speedometers, data of the feature parameter of bridge train-induced deflection can be automatically classified into four cases of train load. Combining the matching relationship of the time coordinate between the non-stationary section of train-induced deflection and the non-stationary section of train-induced acceleration, data of the feature parameter of bridge train-induced acceleration can be automatically classified into four cases of train load. The details of the four cases of train load are indicated in Table 2. Then, the performance index of the bridge can be accurately calculated based on each case of the feature parameter of the bridge train-induced response.

### 5.3. Obtainment and Application of Performance Index for Representation

#### 5.3.1. Dynamic Performance of In-Service Bridges

Regarding the dynamic performance of in-service bridges, we establishes an index of the dynamic performance of the bridge based on the frequency of train-induced forced vibration.

**Figure 11:**
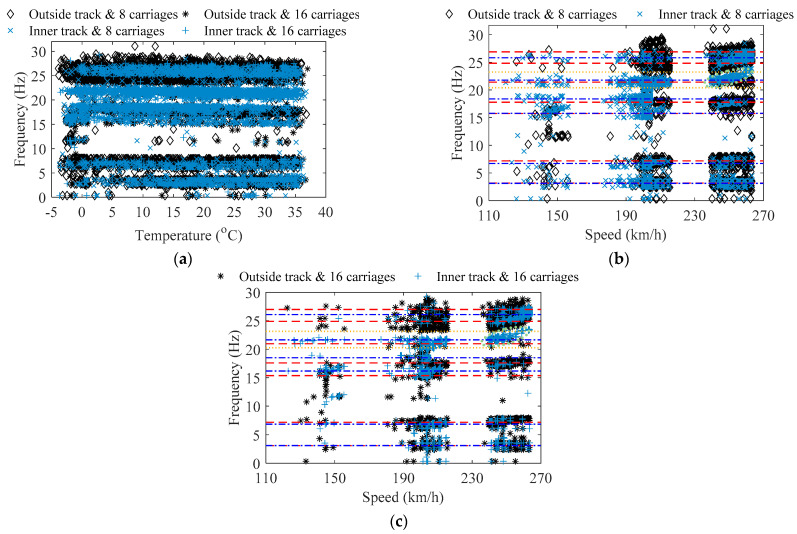
Frequency stability of the train-induced forced vibration: (**a**) influence of temperature; (**b**) influence of train speed in the cases of 8 carriages; (**c**) influence of train speed in the cases of 16 carriages.

Figure 11 indicates the frequency value of the peak points of the power density spectrum for each train-induced non-stationary acceleration, namely the main frequency of train-induced forced vibration, as well as its corresponding data of temperature and train speed. As indicated in Figure 11, in each case of train load, the identified frequencies of train-induced forced vibration are concentrated in 6 to 7 main frequency bands, and the data distribution of each band of frequency is relatively discrete, which is due to the randomness of the train carriage′s weights, track irregularities, difference in parameters of the train′s suspension system or any other variation; this phenomenon is similar to the characteristics of frequencies of traffic-induced forced vibration in the study of Ref. [52]. Accurate identification of each dense mode of the train-induced forced vibration of the bridge may be meaningless, because each variation of the dynamic parameters in the train–bridge system make the frequency of train-induced forced vibration different; it may be more appropriate to use the concept of data clusters to describe the frequencies of train-induced forced vibration. According to Figure 11a, the frequency of train-induced forced vibration has a slight correlation with temperature; the frequency will slightly decrease as the temperature rises. It should be noted that, as indicated by the dotted circle in Figure 11b,c, in the range of 230–270 km/h and 20.60–23.94 Hz in the cases of the inner track, the frequency of train-induced forced vibration and the train speed presents a nonlinear positive correlation.

Via the method of hierarchical clustering, the data of the frequency of train-induced forced vibration in Figure 11 is clustered, and the data clusters of the main frequency of train-induced forced vibration in the four cases of train load can be obtained. Considering that the cases of the inner track show a correlation between the frequency of train-induced forced vibration and train speed, the clustering of the cases of the inner track uses the two-dimension data of frequency–train speed, while the clustering of the cases of the outside track uses the one-dimension data of frequency. Since temperature has little influence on the frequency of train-induced forced vibration, the two-dimension data of frequency–temperature are not used in hierarchical clustering here; the clustering result of the one-dimension data of frequency and the clustering result of the two-dimension data of frequency–temperature are the same. The depth of the hierarchical clustering of the cases of the inner track is set to 12, and the depth of the hierarchical clustering of the cases of the outside track is set to 10. In order to obtain better results of clustering and reduce the influence of train speed on clustering, the data value for train speed used for clustering is uniformly divided by 500. It should be noted that the clustering parameters of hierarchical clustering require calibration for different bridges.

In Table 3 and Figure 11b,c, the estimated value of the centroid for the main orders of the frequency of train-induced forced vibration after hierarchical clustering is indicated; the dashed line is the centroid of the main orders of the frequency of train-induced forced vibration from the cases of the outside track, while the dash-dotted line is the centroid of the main orders of the frequency of train-induced forced vibration from the cases of the inner track. The centroid can be calculated from each cluster by Equation (19). Table 4 indicates the estimated value of the band range for the main orders of the frequency of train-induced forced vibration after hierarchical clustering. The band range of each cluster takes value of the 0.05 and 0.95 quantile of the cumulative distribution function (CDF) of the Gaussian from each cluster. The estimated value of the bands for the fifth order of the frequency of train-induced forced vibration from the cases of the inner track is also shown in Figure 11b,c by the dotted line; the data in the corresponding cluster is in the range of 19.60–23.94 Hz. As indicated in Figure 11 and Table 3 and Table 4, it can be found that the centroid of the obtained data clusters can effectively represent the average characteristic of the main frequencies of train-induced forced vibration; the band range can effectively envelop most data, including most of the frequency data with the nonlinear correlation of train speed in Figure 11b,c (it cannot be achieved by the *k*-means clustering). If the CDF quantile is closer to 0 and 1, each order′s band range can envelop more of the data in each cluster, but the data of the outlier should be eliminated in engineering. The data of train-induced forced vibration frequency are relatively discrete and reflect the randomness and variability from the excitation of train loads; these data cannot be neglected. The results and obtaining process of the whole method can provide references for the design, operation, and maintenance of long-span cable-stayed railway–highway combined or high-speed-railway bridges.

#### 5.3.2. Deformation Performance of In-Service Bridges

Regarding the deformation performance of in-service bridges, we established the index of the deformation performance of the bridge based on the distribution of the amplitude of train-induced deflection.

**Figure 12:**
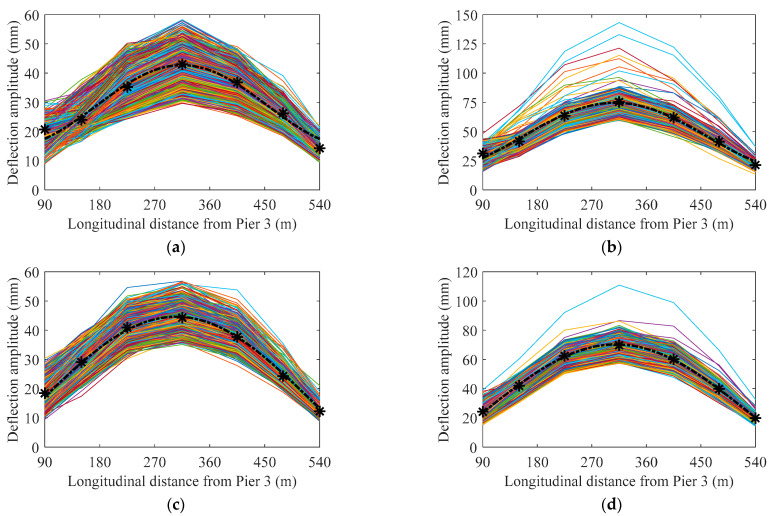
Distribution of train-induced deflection amplitude in each traversing of trains: (**a**) outside track and 8 carriages; (**b**) outside track and 16 carriages; (**c**) inner track and 8 carriages; (**d**) inner track 16 carriages.

Based on the classified results of the train-induced deflection amplitude data in the four cases of train load, and the one-to-one matching relationship between several deflection sensors along the girder of the main span, Figure 12 indicates the distribution lines of train-induced deflection amplitude along the main span of the bridge based on one year′s data of train-induced deflection amplitude in the four cases of train load. The fitting curve of the distribution for the centroid of the data from each sensor is established using the Fourier series:

(21) $$DC_{\mathrm{tdf}}(x)=b_{0}/2+\sum_{i=1}^{\infty }\left[b_{i}\cos(\omega_{f}x)+c_{i}\sin(\omega_{f}x)\right]$$

where *b*_0_, *b_i_*, *c_i_*, and *ω*_f_ are the fitting parameters. All the fitting curves use the first order *Fourier* series in this paper. The calculation of fitting parameters adopts the least squares method. The centroid of the data from each sensor is shown as the star icon in Figure 12, and the fitting curve of the distribution for the centroid of the data from each sensor is shown as the dash-dotted curve in Figure 12. The number of traversing trains in Figure 12a–d are 3792, 1798, 3283, and 1601, respectively.

**Figure 13:**
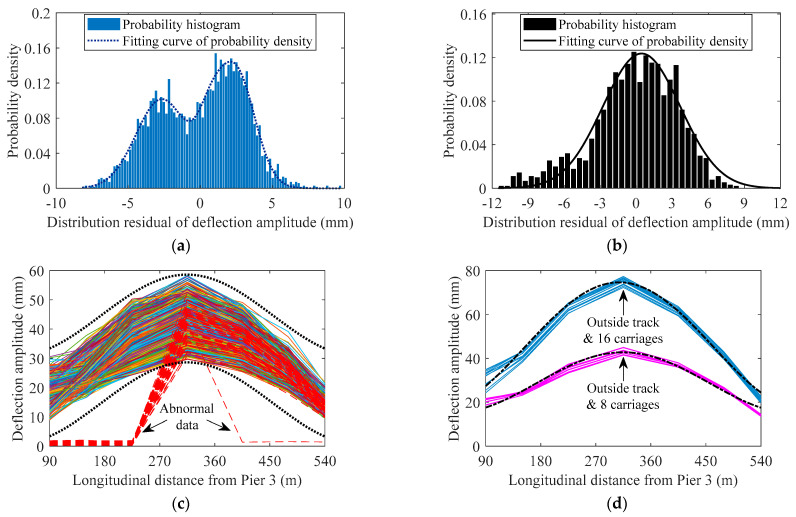
Characteristics and discussion of the distribution of train-induced deflection amplitude: (**a**) probability density analysis of typical *DR*_td_ of the outside track and 8 carriages case; (**b**) probability density analysis of typical *DR*_td_ of the outside track and 16 carriages case; (**c**) normal range of the outside track and 8 carriages case; (**d**) *D*C_tdf_ of one year and the distribution of the centroid from each sensor′s 12 single-month data.

After the *DC*_tdf_ of the bridge main span from each deflection sensor in a certain period (in this paper, it is one year) is obtained, the distribution residual of deflection amplitude in each traversing of trains should be analyzed to represent the train loads′ randomness and variability. For each traversing of trains, the distribution residual between the multiple monitoring sections′ train-induced deflection amplitude and the *DC*_tdf_ can be expressed as

(22) $$DR_{\mathrm{td}}=\frac{1}{S}\sum_{j=1}^{S}\left(AMD_{j}-DC_{\mathrm{tdf}j}\right)$$

where *AMD_i_* denotes the amplitude of the measured train-induced deflection data of the *j*th monitoring section in each traversing of the trains, *D*C_tdf*i*_ denotes the value of the fitting curve of Equation (21) at the location of the *j*th monitoring section, *S* denotes the number of used deflection monitoring sections. Figure 13a,b indicates the fitting function of the probability density of *DR*_td_ using the mixture Gaussian distribution [34]:

(23) $$f(x)=\sum_{i=1}^{l}\varphi_{i}\frac{1}{\sqrt{2\pi }\sigma_{i}}\exp(-\frac{{\left(x-\mu_{i}\right)}^{2}}{2\sigma_{i}^{2}})$$

where *μ_i_*, *σ_i_*, and *φ_i_* denote the mean, standard deviation, and combined weight of each degree of single Gaussian distribution, respectively. As indicated in Figure 13a,b, the *DR*_td_ for the outside track and 8 carriages case obeys the second-order mixture Gaussian distribution, and the *DR*_td_ for the outside track and 16 carriages case obeys the first order mixture Gaussian distribution. The *DR*_td_ for the two cases of the inner track also obeys the first-order mixture Gaussian distribution; it is not presented here.

Based on *D*C_tdf_ and a certain quantile of the CDF of *DR*_td_, the normal range of the distribution of the train-induced deflection amplitude during the bridge operation can be determined. Taking the outside track and 8 carriages case as an example (as indicated in Figure 13c), the upper threshold of the normal range equals the *D*C_tdf_ plus the 0.95 quantile of the CDF of *DR*_td_ plus three times the standard deviation of *DR*_td_, while the lower threshold equals the *D*C_tdf_ plus the 0.05 quantile of the CDF of *DR*_td_ minus three times the standard deviation of *DR*_td_. Here, the multiple of three of the standard deviation can be changed according to different bridges or different deterioration states of bridges; it needs to be dynamically updated when using the proposed method. The calculated upper and lower thresholds (dotted curves in Figure 13c) of the normal range of the distribution of the train-induced deflection amplitude during the bridge operation can be used to detect the abnormal signals (data missing, etc.) or extreme traffic load. For example, dashed lines in Figure 13c present the characteristics of the distribution of train-induced deflection amplitude under the situation that part of sensors′ data are missing and part of sensors′ data are collected normally; the abnormal data is out of the lower threshold of the normal range.

Due to the randomness, variability, and uncertainty of traffic loads, the threshold of the normal range of the distribution of the train-induced deflection amplitude cannot accurately and effectively represent the deformation performance of the bridge. Compared with the threshold of the normal range, the *DC*_tdf_ is more suitable as a performance index of bridge long-term deformation. Based on Equation (14), the *DC*_tdf_ (corresponding to the *A*_sv_ in Equation (14)) will increase with the decrease of the stiffness of the bridge girder. In addition, *DC*_tdf_ can avoid the dilemma that the raw data of measured deflection cannot find a starting point (the baseline) of the stress-free state. Hence, we believe that the *DC*_tdf_ is a suitable index to represent the deformation performance of the bridge. Figure 13d indicates the comparison between the *DC*_tdf_ of one year and the distribution of centroid from each sensor′s 12 single-month data; it can be found that the lines of the distribution of the centroid of 12 months surround the *DC*_tdf_ of one year in the two cases of the outside track; the two cases of the inner track have the same characteristic, so it is not presented here. Based on the analysis of Figure 5a, Figure 6a and Figure 13d, the robustness of *DC*_tdf_ for the ambient temperature and long-term period is validated. Hence, based on the foregoing analysis, the *DC*_tdf_ can be used for the representation index of the long-term deterioration of the deformation performance of the bridge, which serves for the evaluation, early-warning, operation, and maintenance works of long-span cable-stayed railway–highway combined or high-speed-railway bridges.

In the process of monitoring, operation, and maintenance of the bridge, the time-varying index *DC*_tdf_ can be calculated using the monitoring data of the bridge from the current moment to a period in the past (e.g., one year). According to the principle of mechanics, as the stiffness of a beam decreases, its deflection will be higher under the same live load. The daily weight and speed of passenger high-speed trains on the bridges do not change much. When *DC*_tdf_ decreases slowly, it indicates that the deformation performance of the whole bridge (or the whole girder) deteriorates in the operation. The maximum point of *DC*_tdf_ plus the annual change amplitude of temperature-induced deflection can be used the early-warning index for the danger of a bridge. When the calculated centroid of the train-induced deflection amplitude in one or more monitoring section (as the star icon in Figure 12) shows a great change in the distance from *DC*_tdf_, it means that a certain amount of the redistribution on the internal force of the structure has occurred in the bridge (that is, there is a certain amount of the local damage in the bridge). Deterioration and redistribution require long-term data or experiments to verify and calibrate; we only present the method in this study.

## 6. Conclusions

Aiming at the quick quantification problem of the real-time performance of long-span bridges, this paper proposes a representation method of in-service performance using a case study of a cable-stayed railway–highway combined bridge. The process of representation integrates theories of signal processing, structural dynamics, data clustering, and statistical modeling and can obtain accurate and effective indexes of bridge performance from the massive data of monitoring, which is filled with randomness and variability. The whole process of the proposed method verifies the feasibility of the representation of bridge in-service performance from ‘train-induced response at every point of sampling frequency’ to ‘feature parameters of response in each traversing of trains’ to ‘long term indexes of performance’. The main contributions of this study are as follows.

1. After the normalization of temperature′s effect on the deflection data, train-induced non-stationary sections of deflection and acceleration are automatically distinguished and extracted based on the characteristics of the time series signal of live load-induced deflection, train speed, and acceleration.
2. Derived calculational theory proves that the train-induced deflection amplitude under 16 carriages train is larger than that under 8 carriages. This provides knowledge for the clustering of feature parameters of train-induced response for different cases of train load.
3. Clusters of the main orders of the frequency of train-induced forced vibration are obtained via hierarchical clustering. Their stability for temperature and train speed are discussed and validated. The centroid and band range of each cluster are calculated as the index to represent the current dynamic performance of the bridge.
4. Distribution of the centroid of the train-induced deflection amplitude of the bridge main span driven by the data of multiple sensors is fitted as the index, which is used to represent the long-term deterioration of the deformation performance of the bridge. The statistical threshold of the normal range of the distribution of train-induced deflection amplitude is determined to detect and clean the data with abnormal signals.

## Figures and Tables

**Table 1:** Sensing details for multiple types of sensors.

Sensor Type	Sampling Frequency	Sensing Content	Data Unit
Hydrostatic leveling instrument	5 Hz	Deflection (Displacement)	mm
Accelerometer	100 Hz	Acceleration	mm/s^2^
Atmospheric thermometer	1 Hz	Temperature	°C
Radar speedometer	10 Hz	Speed	km/h

**Table 2:** Four cases of train load for data classification.

Case 1	Case 2	Case 3	Case 4
Outside track and 8 carriages	Outside track and 16 carriages	Inner track and 8 carriages	Inner track and 16 carriages

**Table 3:** Centroid estimation for the main orders of the frequency of train-induced forced vibration.

Case	Centroid (Unit: Hz)						
	1st Order	2nd Order	3rd Order	4th Order	5th Order	6th Order	7th Order
Outside track and 8 carriages	3.18	7.19	15.74	17.78	21.41	24.82	26.89
Outside track and 16 carriages	3.09	7.20	15.37	17.59	20.96	24.90	26.99
Inner track and 8 carriages	3.12	6.71	15.77	18.37	21.77	25.81	/
Inner track and 16 carriages	3.11	6.86	16.17	18.51	21.66	26.09	/

**Table 4:** Band range estimation for the main orders of the frequency of train-induced forced vibration.

Case	Item	Lower and Upper Limits of the Band Range (Unit: Hz)						
		1st Order	2nd Order	3rd Order	4th Order	5th Order	6th Order	7th Order
Outside track and 8 carriages	lower	1.75	6.28	14.69	17.21	20.56	23.92	25.92
	upper	4.61	8.10	16.79	18.37	22.27	25.73	27.87
Outside track and 16 carriages	lower	1.56	6.32	14.60	16.79	19.98	23.72	26.10
	upper	4.63	8.09	16.14	18.40	21.91	26.07	27.89
Inner track and 8 carriages	lower	1.82	5.69	14.69	17.60	20.60	24.84	/
	upper	4.42	7.73	16.86	19.11	22.94	26.79	/
Inner track and 16 carriages	lower	1.32	6.05	15.10	17.87	20.46	24.80	/
	upper	4.91	7.68	17.25	19.16	22.85	27.39	/

## Data Availability

The raw data supporting the conclusions of this article will be made available by the corresponding author upon reasonable request.
